# *Insertion Sequences *show diverse recent activities in Cyanobacteria and Archaea

**DOI:** 10.1186/1471-2164-9-36

**Published:** 2008-01-24

**Authors:** Fengfeng Zhou, Victor Olman, Ying Xu

**Affiliations:** 1 Computational Systems Biology Laboratory, Department of Biochemistry and Molecular Biology and Institute of Bioinformatics, University of Georgia, Athens, GA 30602, USA

## Abstract

**Background:**

Mobile genetic elements (MGEs) play an essential role in genome rearrangement and evolution, and are widely used as an important genetic tool.

**Results:**

In this article, we present genetic maps of recently active *Insertion Sequence *(IS) elements, the simplest form of MGEs, for all sequenced cyanobacteria and archaea, predicted based on the previously identified ~1,500 IS elements. Our predicted IS maps are consistent with the NCBI annotations of the IS elements. By linking the predicted IS elements to various characteristics of the organisms under study and the organism's living conditions, we found that (a) the activities of IS elements heavily depend on the environments where the host organisms live; (b) the number of recently active IS elements in a genome tends to increase with the genome size; (c) the flanking regions of the recently active IS elements are significantly enriched with genes encoding DNA binding factors, transporters and enzymes; and (d) IS movements show no tendency to disrupt operonic structures.

**Conclusion:**

This is the first genome-scale maps of IS elements with detailed structural information on the sequence level. These genetic maps of recently active IS elements and the several interesting observations would help to improve our understanding of how IS elements proliferate and how they are involved in the evolution of the host genomes.

## Background

Mobile genetic elements (MGEs) can move themselves within a genome and between genomes. They play key roles in modification of gene expression patterns by generating insertion mutations [1-3] and in genome rearrangement and evolution through homologous recombination [4-6]. Some of them, such as Tn*5 *in *E. coli *[7] and *Salmonella typhimirium *[7,8], have been extensively used to mediate insertional mutagenesis to perform genetic studies. The simplest form of MGE is the *insertion sequence *(IS) element, which usually encodes only a transposase [9]. IS elements are widely distributed in eubacterial and archaeal domains [9-11] and more than 1,500 different IS elements have been identified as of now [12].

**Figure 1:**
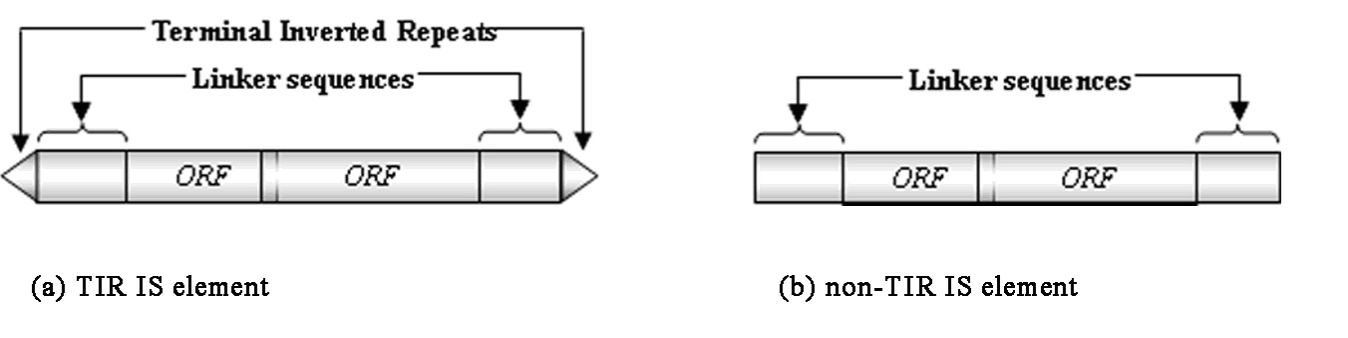
Conformation of a typical (a) TIR or (b) non-TIR IS element.

IS elements are usually organized compactly, most of which span 700 to 3,500 bps [see Additional file 1, Table S1]. Many IS elements have only one ORF, encoding a transposase, while others have more than one ORF [9,13,14]. For an IS element with more than one ORF, the first (upstream) ORF encodes a DNA recognition domain, while the second one, overlapping the first one, encodes the catalytic domain. Typically a so-called *slippery codon *in a heptamer nucleotide sequence X XXY YYZ in the overlapped region between the two ORFs incurs a -1 translational frame-shift, providing a regulatory mechanism for the cell to express either a DNA binding domain or a transposase. One of the most commonly observed example in IS elements is A AAA AAG, which occurs in IS*2*, IS*150*, IS*222*, IS*861*, IS*895*, IS*904 *and IS*1133 *[15]. There are two classes of IS elements, i.e. TIR (*t*erminal *i*nverted *r*epeats) IS elements and non-TIR IS elements. A TIR IS element carries a pair of (partially conserved) inverted repeats at the two termini of an IS for cleavage and binding of the transposase, as shown in Figure 1 (a), while a non-TIR IS element (Figure 1 (b)) does not harbor significant signals around its termini. Our current knowledge is still limited about how an non-TIR IS element is recognized and cleaved by its encoded transposase [9]. The regions between the two termini but outside the ORFs are called *linker sequences *(Figure 1). The ~1,500 known IS elements in *ISfinder *[12], the most comprehensive IS database, are categorized into 20 families and some of them are further categorized into 27 groups [12] based on the similarities of their genetic organizations, transposase sequences and the TIR signals [9,13] [see Additional file 1, Table S1]. Currently all but one IS family (the IS200/IS605 family) are made of TIR IS elements.

While some IS elements have proliferated across multiple eubacterial and archaeal genera, many others were only found in a few closely related organisms. Currently only ~220 out of the ~1,500 IS elements in *ISfinder *[12] are reported to appear in more than one genome, and the general distributions of IS elements across a genome or multiple genomes are not well understood. Hence it is scientifically interesting and important to study the distributions of IS elements across prokaryotic genomes to understand how IS elements proliferate and promote the evolution of their host genomes [16,17].

Previous studies on characterization of IS elements, or transposable elements in general [18-25], have been mostly focused on a few transposable element families rather than attempting to understand their genome-scale distributions. Typically, these computational studies identify transposable elements using one of the following approaches or their combination: (a) identification of transposable elements based on sequence similarity search against known transposons [18,19,22,26,27], (b) identification of short TIRs or long terminal repeats flanking the predicted coding regions of candidate transposons [26,28-31], and (c) identification of all the insertion events through alignments of multiple closely related genomes to determine the inserted regions associated with other features such as the ones in (a) and (b) [32]. We believe that only through identification of all the known IS elements at a genome scale could we possibly discover some of the hidden governing rules about IS elements and their distributions.

In this paper we have investigated the endogenous characteristics of each group of IS elements, as defined in *ISfinder *[12], and built a sequence profile for each of them. Then we present genome-scale maps of predicted IS elements, based on the known elements in *ISfinder*, across cyanobacteria, one subgroup of eubacteria, and all the sequenced archaea along with some observations derived from these large-scale IS maps. In terms of prediction accuracy, we have demonstrated that our method performs better on cyanobacteria and archaea than the only available large-scale IS prediction program, IScan [33].

The focus in this study is on the *recently active *IS elements (raIS), which are defined as IS elements with multiple copies of highly similar sequences in the same genome [34].

## Results and Discussions

### Profiles for IS groups

**Figure 2:**
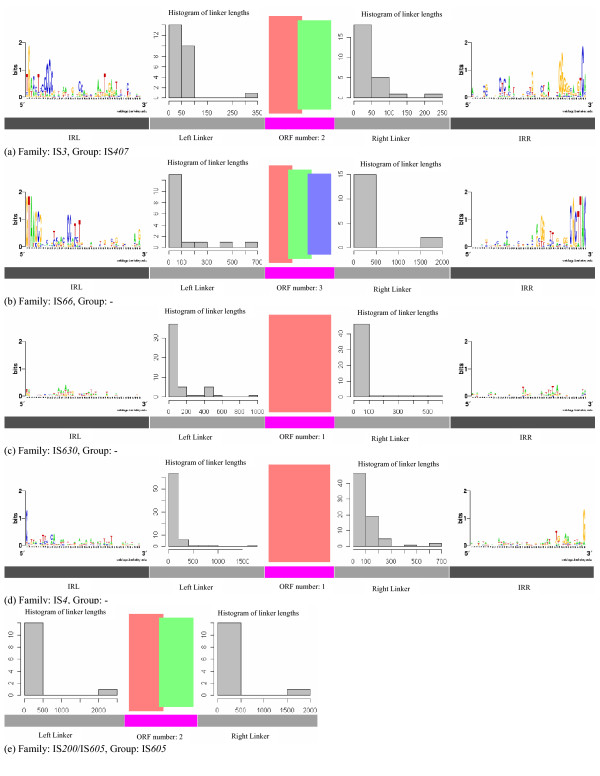
**Schematic illustrations of IS group profiles**. For each IS group, the ORFs of an IS element in this group are represented as boxes with different colors in the middle, the lengths of left and right linker sequences of the IS elements are illustrated as histograms, and the conservation of their TIR signals, if there are any, are shown by a sequence logo. (a) IS407 group of IS3 family, (b) "-" group of IS66 family, (c) "-" group of IS630 family, (d) "-" group of IS4 family and (e) IS605 group of IS200/IS605 family. Please refer to Supplementary Figure S1 for illustrations of all the profiles.

While the coding regions have been extensively used to identify IS elements, the TIR signals and the lengths of linker sequences of IS elements, though highly conserved for some IS groups, have not been used much for prediction of IS elements. For these groups, the TIR signals of the IS elements in each group usually contain a conserved motif, as shown in Figure 2 (a) and 2 (b). Other IS groups may not have conserved sequence motifs as shown in Figure 2 (c) and 2 (d). The similarity among the TIR signals was measured using a position weight matrix (PWM) constructed from the TIR signals of known IS elements in the same IS group [35], and the profiles of non-TIR IS elements do not include the TIR signals (see Figure 2 (e)).

The linker sequences of IS elements in the same group tend to have similar lengths, as shown in Figure 2, and could be used to help identify IS elements as well. For example, although the TIR signals of IS elements in the "-" group of the IS*630 *family are too weak to be recognized, both the 5' and 3' linker sequences generally have conserved sequence lengths in most of the cases (Figure 2 (c)). (For convenience, we define a group "-" for each IS family to accommodate the IS elements with no group information in that family in the ISfinder database. So there are 47 IS groups in total, including the "-" group for each of the 20 IS families.)

We have built a sequence profile for each IS group in *ISfinder *based on the coding sequences, the linker sequences and the TIR signals, if there are any. Additional file 2, Figure S1 shows the sequence profiles for all the 36 IS groups with at least 10 members.

### Overview of raIS maps

We chose cyanobacteria, a very diverse group of bacteria that inhabit a wide range of living environments [36], and archaea as the target organisms in our study, and have applied our Insertion Sequence Annotation (ISA) system to the genome sequences of the 50 selected organisms (19 cyanobacteria and 31 archaea). All the raIS maps of the cyanobacteria and archaea are provided in a database that is accessible through an easy-to-use web interface [37]. 45 IS elements are predicted to be recently active in the 19 cyanobacteria, and they cover 11 IS families, while 104 IS elements from 12 IS families are predicted to be recently active in the 31 archaeal genomes.

Comparing our predicted IS elements to the NCBI annotations, we found that our prediction covers essentially all the raIS elements annotated by NCBI. The few missing ones are mostly due to the fact that these IS elements have not been active enough recently to have two highly conserved copies in the host genomes, as shown in column I2 of Additional file 1, Table S2. The remaining missing ones are annotated as putative transposases or IS elements that have not been deposited in *ISfinder *(column *IPuative *and *INovel *of Additional file 1, Table S2). The percentage of the NCBI IS genes that are missed by our prediction is lower than 10% for all the 50 genomes except for three, *Anabaena variabilis *ATCC29413 (19.32%), *Pyrobaculum islandicum *DSM 4184 (21.43%) and *Sulfolobus solfataricus *(15.40%). Many of them are misannotated and should be IS elements that have not been deposited in *ISfinder*. For example, almost all such genes in *Anabaena variabilis *ATCC29413 are annotated as transposases of IS*4 *from the IS*4 *family, but the *E-value *of BlastP search between them and IS elements from IS*4 *family is greater than 9.8.

Many IS related genes are annotated as *putative transposases *by NCBI while our prediction provides more detailed information for most of these IS elements (column *APutative *in Additional file 1, Table S2). In addition, our prediction also finds many IS genes that were not recognized by NCBI, and almost all of them were annotated as hypothetical proteins by NCBI (column *ANovel *and *ANovePutative *of Additional file 1, Table S2).

We have further compared our IS prediction with the prediction by the IScan program [33], the only available prediction program on the Internet, on a test set consisting of IS elements from 20 IS families from *ISfinder*, whose TIR signals and protein sequences have been curated by Wagner et al [33]. On this test set, IScan did not find any IS elements in cyanobacteria or archaea while our prediction results are summaried in Additional file 1, Table S2. This low prediction sensitivity might be due to the high specificity set by the IScan program in identification of the TIR signals. It should be noted that for numerous groups in *ISfinder*, the IS elements from the same group may not have conserved TIR signals, as shown in Figure 2 as well as in Additional file 2, Figure S1.

Another comparison we made is with a data set, manually curated by Filee et al [11], which surveyed all the known IS elements in archaeal genomes. Their results regarding which IS group has presence in archaea are given in Additional file 1, Table S1. Our prediction agrees well with the results by Filee et al except for 11 IS elements (IS*Hma4*, IS*H4*, IS*H50*, IS*Nph2*, IS*Mma22*, IS*Hma12*, IS*C1041*, IS*Mac15*, IS*Mbu9*, IS*Arch5*, IS*Mac21 *and IS*Mbu4*), which were not predicted by our program Among these 11 elements, one element, IS*C1041*, is only observed in *S. solfataricus *MT-4, whose genome has not been sequenced, and all other 10 elements have only one or two full copies in the host genomes, below our cutoff for raIS element prediction.

We have carefully analyzed our predicted raIS elements in the 50 genomes. We observed that in our raIS maps, raIS elements tend to be clustered together in the host genomes. As shown in Additional file 1, Table S3 where each genome is partitioned into 100 kbp windows, the numbers of raIS elements within these windows vary substantially. Some windows, say in *Trichodesmium erythraeum *IMS101, could have as many as 18 raIS elements, while at least 50% of the windows contain at most 1 raIS element. This observation holds for the majority of the 50 genomes under study. This could be due to the fact that IS elements tend to insert their copies into the so called *hotspot *regions, where new IS insertions may be less lethal to the organism [10]. Another possible explanation could be that the nearby genomic region of a raIS copy may have similar accessibility for the enzymes involved in transposition.

We find that raIS elements of different IS groups clearly have different distributions. 28 out of 47 (59.57%) IS groups show recent activities in cyanobacteria or archaea, and the two most widely deployed ones (the groups "-" of families IS4 and IS630) appeared in at least 9 organisms [see Additional file 1, Table S1]. On the other hand, there are 19 IS groups (40.43%) that were silent in both cyanobacteria and archaea. We also found that there are 6 and 11 IS groups that appear only in cyanobacteria and archaea, respectively [see Additional file 1, Table S1]. Similar numbers are also observed at the IS family level.

We have further compared the distribution of each IS group in our annotations and in the *ISfinder *database, as shown in Additional file 1, Table S1. Four IS groups are identified to be in cyanobacteria by *ISfinder*, but they seem to have no recent transposition activities, since there were no predicted raIS elements from them in cyanobacteria. Similarly, there are 7 such IS groups in archaea. In addition, we have discovered recently active members from 6 IS groups that were not proposed by the *ISfinder *database to appear in cyanobacteria, while no such a group was identified in archaea.

It is worth mentioning that a single-copy IS element might also be active in the host genome. For example, a recently invaded IS element in a genome might not have enough time to accumulate more than one copy. But it is difficult to identify the complete structures of these IS elements. This work defines raIS elements as those with two other highly conserved copies in the same genome, and identifies the complete structures of the IS elements with recent activities by comparing the multiple copies of the same element.

### Common characteristics of prokaryotes with large numbers of raIS elements

**Figure 3:**
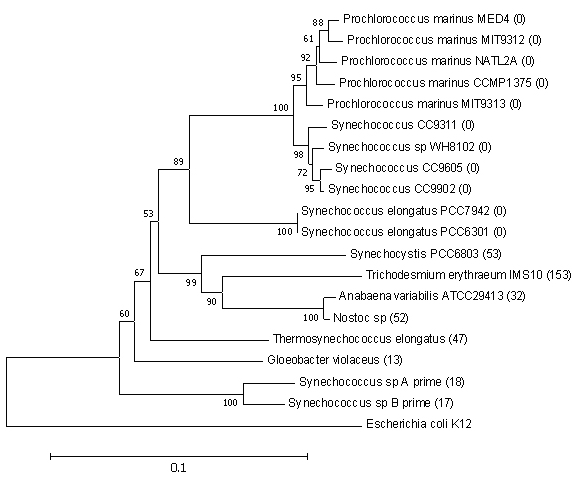
**The bootstrapped neighbor-joining phylogenetic tree of 19 cyanobacteria based on 16S RNA genes**. It is rooted by *E. coli *K12. The number in parentheses after an organism name is the number of annotated raIS elements in that organism. The phylogenetic tree is constructed by MEGA version 3.1 [50].

**Figure 4:**
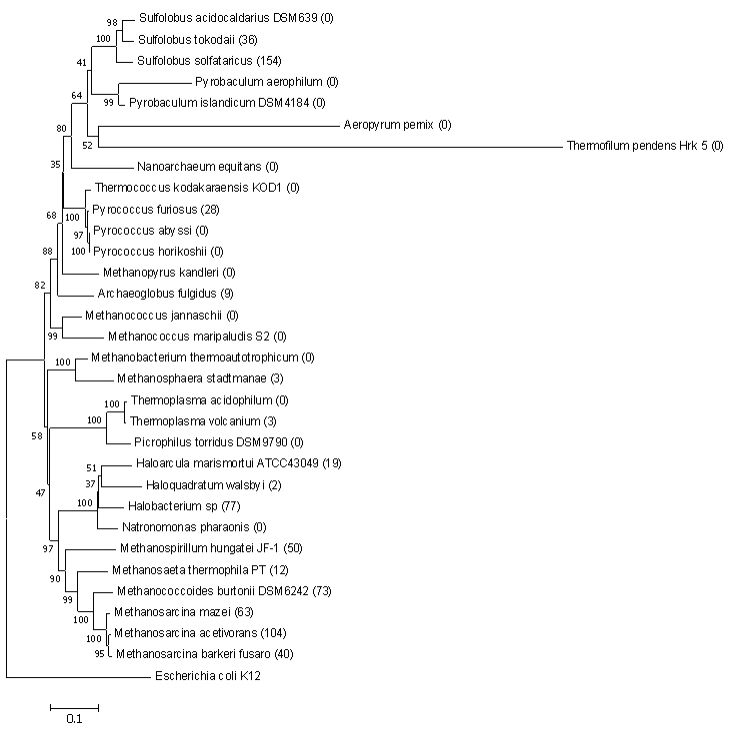
**The bootstrapped neighbor-joining phylogenetic tree of 31 archaea based on 16S RNA genes**. It is rooted by *E. coli *K12. The number in parentheses after an organism name is the number of annotated raIS elements in that organism. The phylogenetic tree is constructed by MEGA version 3.1 [50].

From the predicted raIS maps, we find that the numbers of raIS elements in different cyanobacteria and archaea vary significantly, and many organisms don't harbor any raIS elements, as shown in the two phylogenetic trees for the 19 cyanobacteria and 31 archaea, respectively (Figure 3 and 4). Specifically, no raIS elements are found in *Prochlorococcus *and *Synechococcus*, except for the two newly sequenced strains, *Synechococcus *sp A prime and B prime, while all the other 6 cyanobacteria have very active IS elements, called *raIS-enriched *organisms. We did not observe similar patterns in archaea, possibly due to the very small sampling pool consisting of archaeal genomes with distant relationships. Nevertheless, there are two groups of archaea, *Sulfolobus *and *Methanosarcinales*, which harbor many copies of raIS elements. It is intriguing to learn whether there are any other common characteristics among the raIS-enriched organisms besides the phylogenetic relationships, which may be key factors to the mode and tempo of genome evolution. Clearly the living environment should be one of the first factors to consider.

It was proposed before that IS elements were actively involved in genomic rearrangements when the host organisms were in certain living environments [38,39]. Our result suggests that hot springs seem to be one of the favorite living environments for organisms with active IS elements. Four out of the six organisms retrieved from hot springs are raIS-enriched except for *Sulfolobus acidocaldarius *DSM639 and *Thermofilum pendens *Hrk 5, as shown in Additional file 1, Table S4. Two of the raIS enriched organisms were previously known to harbor active IS elements [40,41], and the two newly sequenced hot spring living *Synechococcus *strains are predicted to have many copies of IS genes by our program as well as by the NCBI database. Another interesting observation is that none of the five archaeal organisms living in thermal vents have raIS elements.

Organisms living in other environments, such as dry land and sewage sludge, are highly diversified in terms of the abundance of raIS elements in their genomes. This may indicate that the living environment might not be the only factor determining activities of IS elements.

**Figure 5:**
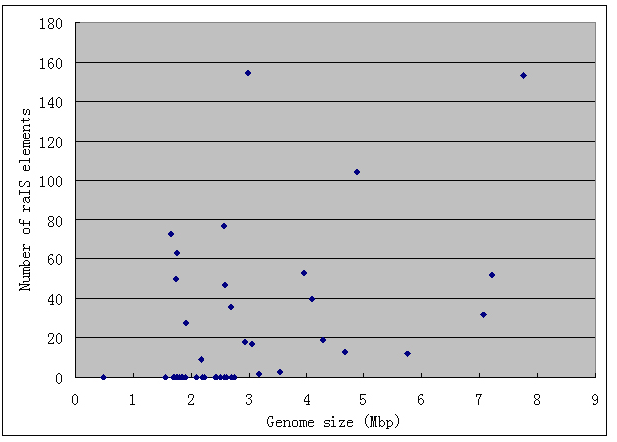
Point plot of genome size and number of raIS elements for each cyanobacteria and archaea.

We have also investigated the correlation between the genome size and the number of raIS elements in a genome. We have used the Spearman's correlation coefficient to check the null hypothesis that the number of raIS elements in a genome is independent of the genome size. As shown in Figure 5, the Spearman's correlation coefficient for our data is 0.5092, and the *P-value *for the null hypothesis (i.e., the two values are not correlated) is 7.95e-5. So the hypothesis is rejected and therefore we conclude that the number of raIS elements in a genome tends to increase with the genome size.

It would be interesting to confirm these observed common characteristics of raIS-enriched organisms on a much larger set of genomes.

### Functions of neighboring genes of raIS elements

Many IS elements could possibly affect the expression patterns of the neighboring genes. For example, the outward directed promoter hexamers inside the TIR signals of many IS elements, such as IS1, IS2, and IS5, may activate the expression of the neighboring genes, if the IS elements are inserted appropriately [9,13].

We have carried out an analysis on the enriched GO functional categories among the neighboring genes of raIS insertions, and calculated a *P-value *for each GO functional category to evaluate the probability that it appears just by chance. Additional file 1, Table S5 shows the enriched GO functional categories with *P-values *< = 0.05 in each organism and their *enrichment ratios *(*ER*), as defined in *Materials and Methods*. While we found that the enriched functions in the neighborhoods of the known IS elements are quite diverse, there are several interesting common patterns in the significantly enriched functions in the neighboring genes of the raIS elements.

We have observed that the neighboring genes of raIS elements are significantly enriched with genes that encode transposases (GO:0004803) in two organisms, i.e. *Cyanobacteria bacterium *Yellowstone A Prime (*ER *= 8.21) and *Sulfolobus solfataricus *(*ER *= 9.3). This might suggest that IS elements tend to insert their copies into the neighboring regions of themselves, since these regions have proved to be safe for receiving transpositions previously. And the mechanism might have contributed to the development of the hotspot regions that received much higher numbers of transpositions than other parts of the same genome, as observed in the above. Besides the transposases (GO:0004803), other DNA binding factors (GO:0003677 and GO:0003702) are also significantly enriched in the neighboring regions of raIS insertions. For example, the enrichment ratio of RNA polymerase II transcription factors (GO:0003702) reaches 19.62 in *Sulfolobus tokodaii*, as shown in Additional file 1, Table S5.

Transporters are another category of genes that are enriched around IS elements in 9 out of 23 organisms (39.13%) with raIS annotations (3.1 ≤ *ER *≤ 35.18), as shown in Additional file 1, Table S5. They are mainly organic acid and amine transporters. Genes encoding different ion transporters are also significantly enriched in the neighbors of raIS elements in *Pyrococcus furiosus *(*ER *= 28.72), *Methanosarcina barkeri fusaro *(*ER *= 35.18), *Methanosaeta thermophila *PT (*ER *= 19.38) and *Thermosynechococcus elongatus *(*ER *= 6.94) [see Additional file 1, Table S5]. As far as we know, this has not been observed and reported in the published literature, and may require further experimental validation.

The flanking regions of some enzymes seem to be another favorite place for IS transposition. The neighboring genes of raIS elements are enriched with transferases in 13 out of the 23 (56.52%) organisms with raIS annotations (2.51 ≤ *ER *≤ 142.35). Specifically, the enrichment ratio of nicotinate nucleotide dimethylbenzimidazole phosphoribosyltransferase (GO:0008939) reaches 142.35 in *Archaeoglobus fulgidus*. Another enzyme tRNA ligase is enriched with ratio 121.14 in the neighbors of raIS elements in *Haloarcula marismortui ATCC 43049*, and two other genomes.

### Transpositions do not prefer inner regions of operons

Insertion of an IS element into an operon might disrupt its structure and hence its function. So we have studied where the predicted raIS elements tend to be with respect to the operonic structures in a genome.

There are ~181 multi-gene operons containing ~454 genes in each genome on average predicted by VIMSS [42]. Other genes are assumed to constitute single-gene operons. Almost all predicted raIS elements form operons with themselves with no other genes, as shown in Additional file 1, Table S2. It's interesting to observe that all raIS elements do not reside in the same operon with their neighboring genes in both cyanobacteria and archaea. One explanation is that an IS element transposed into an operon and separated it into two parts that could be transcribed individually. However since all the raIS elements are not transcribed together with the neighboring genes, the transcription of the genes in these operons, whose internal regions received recent IS transpositions, would have been totally disrupted without the original transcription starting or terminating signals. Such drastic change of operons could possibly be lethal to the organisms. This suggests an alternative explanation that IS elements do not tend to insert into operons.

In summary, raIS insertions do not have the tendency to transpose into the inner regions of operons, which would in general disrupt the operon structures with IS's endogenous transcriptional promoters or terminators.

## Conclusion

We have presented genome-scale maps of recently active IS elements in cyanobacteria and archaea with complete genomes. Based on these maps, we observed that the size of the host genome and the living environment seem to be two important factors to the activity level of IS transposition. Many IS elements seem to prefer inserting into the regions close to the previously transposed IS elements, which might have led to the creation of hotspot regions that harbor many more copies of raIS elements than other regions in the same genomes. IS elements seem also to prefer inserting into neighboring regions of genes encoding other DNA binding factors, transporters and enzymes. It is not observed that an IS element lands into an operon without disrupting the operon structure. And usually the IS elements just avoid inserting into operons.

The genome-scale maps of the raIS elements in this work provide highly informative data for studying the distributions of IS elements and how they incur recombination mutations to the host genomes, which represents the next step of our study.

## Methods

### Datasets

We have manually collected 1,356 IS elements with both sequences and terminal signals from the *ISfinder *database [12] as the templates for our identification of IS elements and map construction in the target genomes. The reason that we did not use some of the ~1,500 IS elements in *ISfinder *is that they are not completely characterized and may not have all the information needed for prediction of IS elements in other genomes.

We chose to investigate the distributions of IS elements in 19 cyanobacteria with complete genome sequences (available in Dec 2006) as the representative group of eubacteria. Since there were only 31 archaea with complete genome sequences (as of Dec 2006), we included all the sequenced archaea in our study. The genome sequences and their annotations were downloaded from the NCBI Genome Project ftp server [43]. The Gene Ontology (GO) annotations of their genes were downloaded from Integr8 [44,45]. Operons for these organisms were predicted using VIMSS [42] with default cutoff score 5.

Since NCBI database provides a comprehensive collection of annotations for each sequenced genome, we chose to investigate the commonalities and differences between our predictions of raIS elements and the NCBI annotated *IS genes*, whose encoded proteins' annotations are done based on their associated keywords such as IS, transposase, or putative transposase.

### IS profiles

Each family or group of IS elements shares some common characteristics in their encoded proteins, their linker lengths and TIR signals if there are any. We provide a measurement for each of these factors, and integrate them using a neural network to predict a region as an IS element.

#### Encoded proteins

Proteins encoded by IS elements from the same IS group are highly similar to each other, while only little or no sequence similarity between the proteins in different IS groups could be observed. Specifically 97.62% of the pairs of all the encoded proteins in different IS groups have lower than 10% sequence conservation, while ~80% of the pairs of encoded proteins in the same IS groups have higher than 10% sequence conservation, as shown in Additional file 2, Figure S2 (a). Similar numbers are observed at the IS family level [see Additional file 2, Figure S2 (b)]. We have used tblastn [46] to map all the proteins encoded by the template IS elements onto the target genomes, using e-5 as the *E-value *cutoff. Some IS elements carry more than one ORF, and we merge the neighboring matching regions into one element if they match the proteins encoded by the same IS template, and reside in a structure that keeps the order and corresponding distance among the encoded proteins of the IS template. Only the elements that have no missing ORFs when compared to the IS templates, called *full copies *of coding regions of the corresponding IS templates, are kept for further analysis.

The maximum E-value of the matching regions of a predicted full copy, denoted as *pIS*, is defined as its overall E-value. The score for this copy is defined as *Score*_*c*_(*PIS*) = -ln(*overall *_*E *- *value*(*pIS*))

#### TIR signals

TIR signals of IS elements in the same IS group tend to be conserved, as shown in Figure 2 and Additional file 2, Figure S1. Due to that IS elements in the same IS group can have different lengths of the TIR signals and they are seldom longer than 50 bps, we calculated a position weight matrix (PWM) for the 50-bp 5' and reverse complementary 3' terminal sequences of the IS elements in each IS group. The coding regions of the predicted full copies are considered as the background.

The score of a 50-bp nucleotide sequence *t *by scanning with a PWM profile *M *is calculated similarly to that in [35]:

$$\begin{array}{l} Score_{TIR}(M,t)=\sum_{i\in I_{IC}}^{50}I_{i}\ln\frac{p[i,t(i)]}{q[t(i)]},\\ I_{i}=\left[\sum_{b\in \{A,C,G,T\}}p(i,b)\ln\frac{p(i,b)}{q(b)}\right]/a,\\ a=\frac{n+1}{n+4}\ln(n+1)-\ln(n+4)-\frac{1}{n+4}\times \sum_{b\in \{A,C,G,T\}}\ln q(b)-\frac{n}{n+4}\ln\underset{b\in \{A,C,G,T\}}{\min}q(b), \end{array}$$

where *t*(*i*) is the base at position *i *of *t*, *p*(*i*, *b*) the relative frequency of base *b *at position *i *in *M*, *q*(*b*) the relative frequency of base *b *occurring in the background, and *n *the number of motifs in *M*. A pseudo-count 1 is added to the frequency of each base at each position in the profile when computing *p*(*i*, *b*). The coefficient *a *is for the normalization purpose so that *I*_*i *_is in the region [0, 1]. Sequence conservation varies significantly in different positions of the 50-bp terminal sequences. So a position *i *∈ {1..50} is included in the subset of index *I*_*IC *_⊂ {1..50}, only if its information content $1+\sum_{b\in \{A,C,G,T\}}p(i,b)\ln p(i,b)/\ln4$ is greater than 0.15. The threshold 0.15 is chosen based on our working experience in this project, and other threshold values for the information content may be chosen for TIR signal recognition possibly with a different level of prediction specificity.

We have retrieved the upstream and downstream flanking sequences of a predicted full copy of a TIR IS element in the target genome with the same lengths as those of the IS template, as illustrated in Additional file 2, Figure S3. The 50-bp regions with maximum scores are predicted as the 5' and 3' TIR signals for this full copy *pIS*, respectively. And the scores are denoted as *Score*_*TIR*_(*pIS*,5) and *Score*_*TIR*_(*pIS*,3), respectively.

#### Linker sequences

We now present a model for scoring the linker sequences of a potential IS element, which has not been much used for IS element prediction previously if any. Based on our preliminary study, we found that only the linker lengths of IS elements in the same IS group may have some discerning power as they tend to be conserved. We have used the following scoring scheme to score the candidate linker sequences for a given IS template. This scoring scheme tends to remove outliers and give higher scores for bins with more compact known data.

Given a group of IS templates, (*IS*_1_, *IS*_2_, ..., *IS*_*n*_), let the lengths of 5' and 3' linker sequences of template *IS*_*i *_be *L*_5_(*i*) and *L*_3_(*i*), respectively. Based on the lengths of 5' linker sequences, the IS elements are equally partitioned into *m *bins, *B*_1_, *B*_2_, ..., and *B*_*m*_, at the interval from $\underset{1\leq k\leq n}{\min}\{L_{5}(k)\}$ to $\left(\underset{1\leq k\leq n}{\max}\{L_{5}(k)\}+1\right)$, where *m *is a user defined integer. Let the number of IS elements in *B*_*j *_be |*B*_*j*_|, and the maximum value in *B*_*j *_be *a*_*j*_. Let $a_{0}=\underset{1\leq k\leq n}{\min}\{L_{5}(k)\}$.

For a predicted IS element *pIS *with 5' linker sequence length *L*_5_, if it belongs to the above IS group and falls in bin *B*_*j *_based on *L*_5_, the score to indicate how similar this predicted element is to the known IS elements in the same group is defined as *Score*_*l*_(*pIS*, *L*_5_) = |*B*_*j*_|/*a*_*j *_- *a*_*j*-1_). The score for the 3' linker sequence with length *L*_3 _is defined as *Score*_*l*_(*pIS*, *L*_3_) similarly.

### IS annotation system (ISA)

We have employed the following procedure to annotate raIS elements in a genome.

Step 1. Predict the full copies of the coding regions of all the known IS templates in *ISfinder*, as described in Section 4.2.1. A region may be predicted as full copies of the coding regions of two IS templates, and only the copy with smaller overall *E-value *will be kept.

Step 2. Predict linker sequences and TIR signals, if there are any, for each full copy from Step 1:

a) Predict TIR signals for each copy of TIR IS elements, using the method outlined in Section 4.2.2. And retrieve the linker sequences between the TIR signals and the coding region.

b) For each predicted full copy of the other IS templates, we find the recently transposed copies, which are defined as having sequence identity higher than 80%, and expand the flanking regions to find the linker sequences that also have higher than 80% sequence identity.

Step 3. Find the *highly conserved *copies, which are defined as having more than 80% sequence identity, of a candidate IS element in the same genome.

Step 4. Calculate the scores for the coding region, the linker sequences and TIR signals of a predicted IS element *pIS *as *Score*_*c*_(*pIS*), *Score*_*l*_(*pIS*, *L*_5_), *Score*_*l*_(*pIS*, *L*_3_), *Score*_*TIR*_(*pIS*,5) and *Score*_*TIR*_(*pIS*,3), respectively.

Step 5. A candidate IS element from the above steps is annotated as a raIS element, if it is predicted as an IS element by the neural network predictor (see Section 4.4), and it has at least two other *highly conserved *copies in the same genome.

The prediction performance of the neural network predictor will be evaluated with 10-fold cross validation in the next section.

To make our annotations of IS elements more informative, we also provide the NCBI genes that overlap our predicted IS elements for each of the predicted raIS elements. An NCBI gene is called the *covering gene *of a raIS element, if their overlapping region is at least 80% of the length of either of them.

The TIR signals were not very conserved even for IS elements of the same IS group, as shown in the schematic illustrations of IS group profiles in Figure 2 and Additional file 2, Figure S1, and some of the raIS elements do not even have predicted TIR signals. The potential TIR signals were retrieved from the terminal sequences of these predicted raIS elements using NCBI blast [46,47].

### 10-fold cross validation of neural network predictor

We mapped the nucleotide sequence of each IS template onto the prokaryotic genome where it was retrieved, and retrieved the copies with at least 80% sequence identities as the positive copies of this IS template. There are 5,344 positive copies of 615 IS elements in total. We constructed a negative copy by randomly choosing a 5,000-bp region without homologs to transposases in a prokaryotic genome, found the best matched transposase to that region using tblastn, and predicted the linker sequences and TIR signals, if there were any, for the negative copy. We constructed the same number of negative copies to that of the positive ones.

We used a neural network implemented in Weka [48] to predict whether a region *pIS *is an IS element based on the three values, *Score*_*c*_(*pIS*), *Score*_*TIR*_(*pIS*,5) +*Score*_*TIR*_(*pIS*,3), and *Score*_*l*_(*pIS*, *L*_5_)+*Score*_*l*_(*pIS*, *L*_3_). A copy of an IS element is considered to be a true positive if it is predicted to be a copy of that IS element or its synonyms and isoforms; otherwise it is a false negative. And a data is considered as a true negative if it is predicted not to be a copy of any IS element; otherwise it is a false positive. Let the numbers of true positives, false negatives, true negatives and false positives of the prediction results of the neural network be TP, FN, TN, and FP, respectively. The following measurements, *sensitivity *(*Sn*) and *specificity *(*Sp*), are used to measure the prediction performance of our neural network predictor:

$$\begin{array}{ccc} Sn=\frac{TP}{TP+FN} & \text{and} & Sp=\frac{TP}{TP+FP} \end{array}.$$

The averaged sensitivity and specificity of 10-fold cross-validation of the neural network over 20 runs are 0.83 and 0.99 respectively. IS elements from the same IS groups could be too similar to be easily distinguished from each other. For example, 542 pairs of IS elements from the same IS groups share at least 90% sequence similarities, although they are not synonyms or isoforms. ~94% of the false negatives are predicted as IS elements belonging to the same IS groups with the true IS elements.

### Functional enrichment analysis

We have investigated whether the neighboring non-raIS genes, within 5,000 bps in distance to the annotated raIS elements in a target genome, are enriched with genes in any particular functional categories according to Gene Ontology (GO). The hypothesis is that the genes occur in the neighborhood of raIS elements by uniform randomly drawing from the pool of all genes in the genome. Let the number of genes in a target genome be *N*, and *n *of them be in a specific functional category ***GO***_*F*_. *M *non-raIS genes in this genome reside within 5,000 bps in distance to the raIS elements, and *m *out of them are in the functional category ***GO***_*F*_. The enrichment ratio of category ***GO***_*F *_is defined as:

$$ER=\frac{m/M}{n/N}$$

The probability of having exactly *i *genes of category ***GO***_*F *_in the *M *non-raIS neighboring genes just by chance could be modeled by a hypergeometric distribution [49]:

$$P(i|N,n,M)=\frac{\left(\frac{n}{i}\right)\left(\frac{N-n}{M-i}\right)}{\left(\frac{N}{M}\right)}.$$

And the *P-value *describing that the functional category ***GO***_*F *_appears at least *m *times in the *M *non-raIS neighboring genes just by chance can be calculated as:

$$p=1-\sum_{i=0}^{m}\frac{\left(\frac{n}{i}\right)\left(\frac{N-n}{M-i}\right)}{\left(\frac{N}{M}\right)}.$$

The above hypothesis would be rejected for the functional category ***GO***_*F *_if its *P-value *is significantly lower. Functional categories with *P-values *< = 0.05 are listed in Additional file 1, Table S5.

## List of abbreviations

MGE, mobile genetic element; IS, insertion sequence; raIS recently active IS element; ISA, insertion sequence annotation; ORF, open reading frame; TIR, terminal inverted repeat; PWM, position weight matrix; ER, enrichment ratio; GO, gene ontology

## Authors' contributions

FZ and YX conceived the study, conducted the experiments and wrote the manuscript. FZ and VO designed the statistical analysis. All authors have read and approved the final manuscript.

## Supplementary Material

Additional File 1 Supplementary Tables S1–S5. Supplementary Table S1 gives the distribution of each IS family and group defined in the database *ISfinder*. Supplementary Table S2 gives the comparison between our predictions and the NCBI annotations. Supplementary Table S3 gives the number of raIS elements in the consecutive windows with length 100 kb for each of the nucleotide sequences of the 50 organisms. Supplementary Table S4 collects the living environments of the 50 organisms. Supplementary Table S5 gives the enrichment analysis of each GO term with P-value <= 0.05. Click here for file

Additional File 2 Supplementary Figures S1–S3. Supplementary Figure S1 shows the schematic profiles of all the 36 IS groups with at least 10 members. Supplementary Figure S2 (a) and (b) compare the conservation percentages between pairs of IS elements at the levels of IS groups and families, respectively. Supplementary Figure S3 shows how to retrieve TIR signals for a predicted full copy of a TIR IS element. Click here for file
