# Supplementary material for: Insertion Sequences show diverse recent activities in Cyanobacteria and Archaea
Source: BMC Genomics. 2008 Jan 24;9:36. doi: 10.1186/1471-2164-9-36 (PMC2246112; doi:10.1186/1471-2164-9-36)

**Supplementary figure S1.** Profiles of IS groups. For each IS group, the ORFs of an IS element in this group are represented as boxes with different colors in the middle, the lengths of left and right linker sequences of the IS elements are illustrated as histograms, and the conservation of their TIR signals, if there are any, are shown by a sequence logo. We categorized the elements with no further group information in an IS family as group “-“ of that family. The logo of IR signals for each IS group is generated by [WebLogo](http://weblogo.berkeley.edu/), and the height of each nucleotide is the respective information content. We did not provide the profiles for those groups with less than 10 members. The histogram of linker sequences for each IS family and group is generated by the statistical analysis system [R](http://www.r-project.org/). The above data for each IS family and group was integrated into the final profile by a Perl script based on [GD](http://search.cpan.org/~lds/GD-2.35/) graphics package.

### Family: IS*1*, Group: -


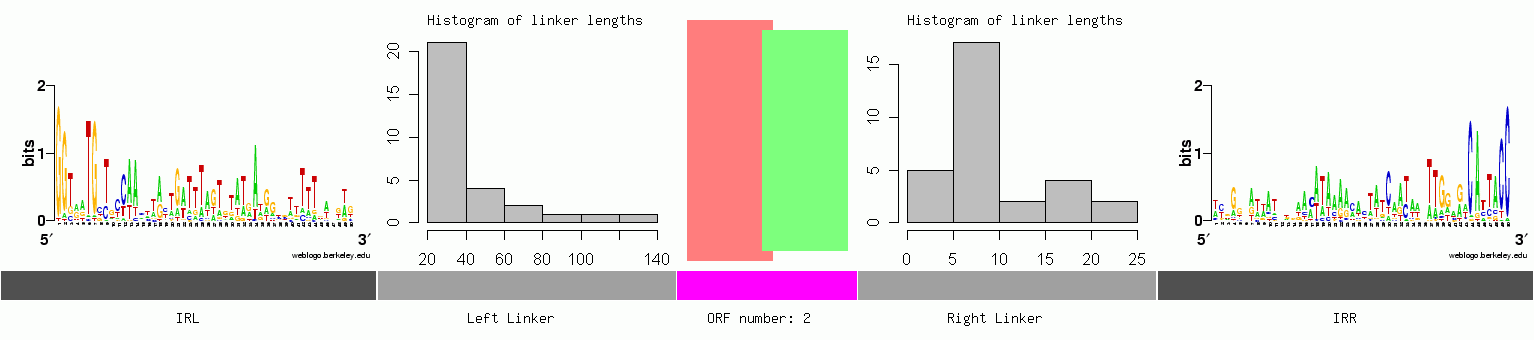


### Family: IS*110*, Group: -


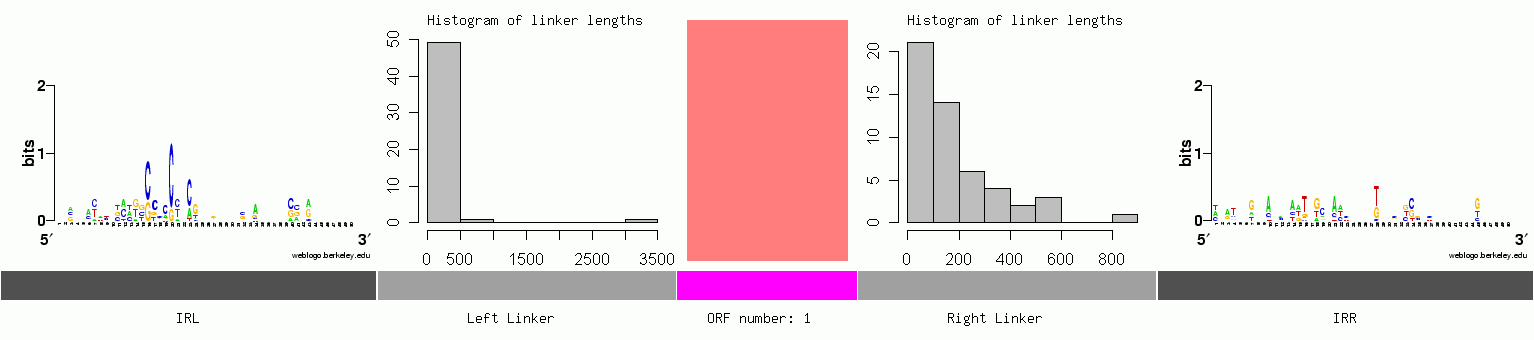


### Family: IS*110*, Group: IS*1111*


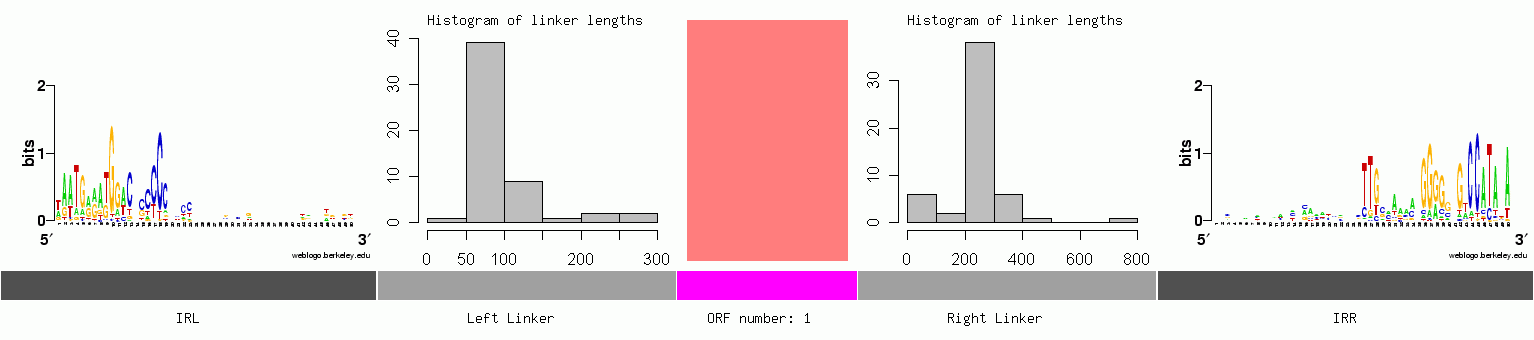


### Family: IS*1380*, Group: -


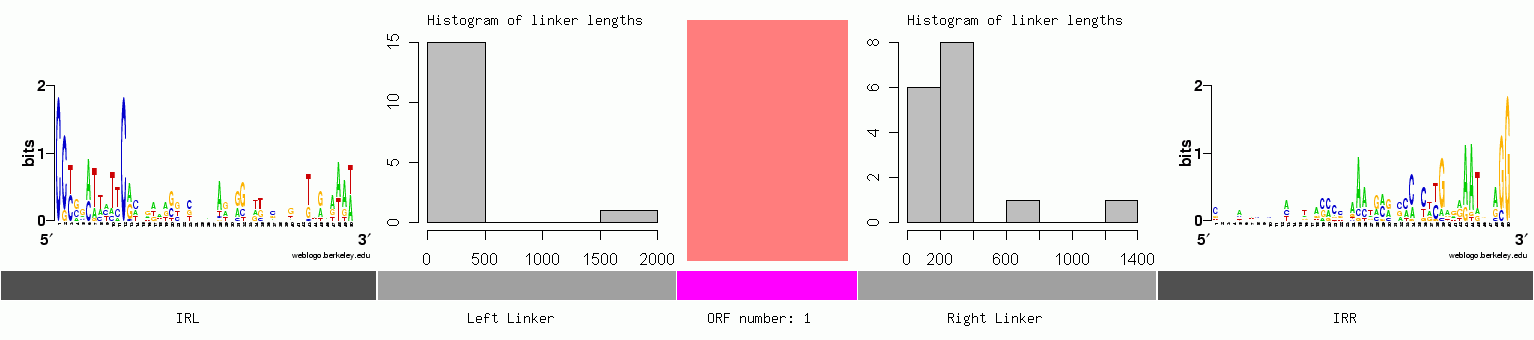


### Family: IS*1595*, Group: -


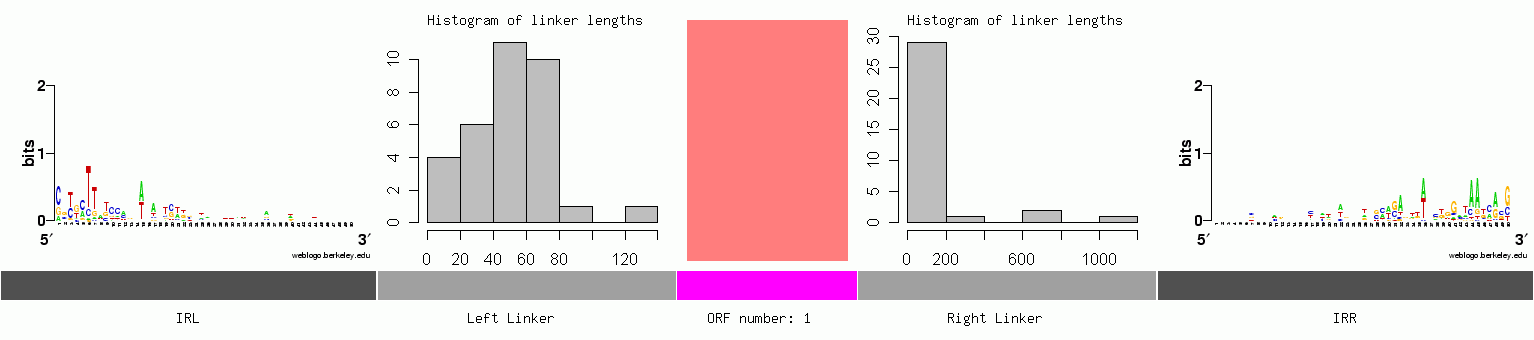


### Family: IS*200*/IS*605*, Group: IS*200*


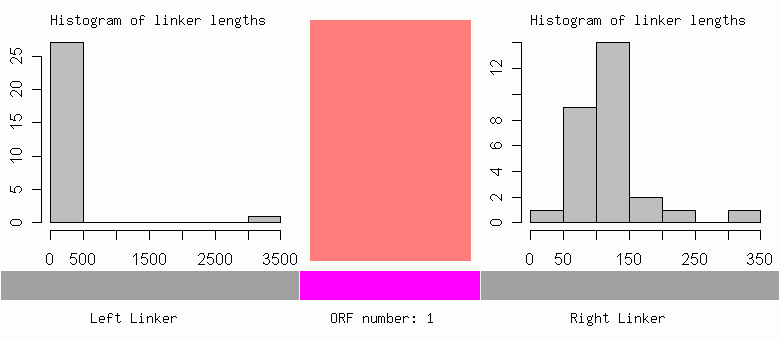


### Family: IS*200*/IS*605*, Group: IS*605*


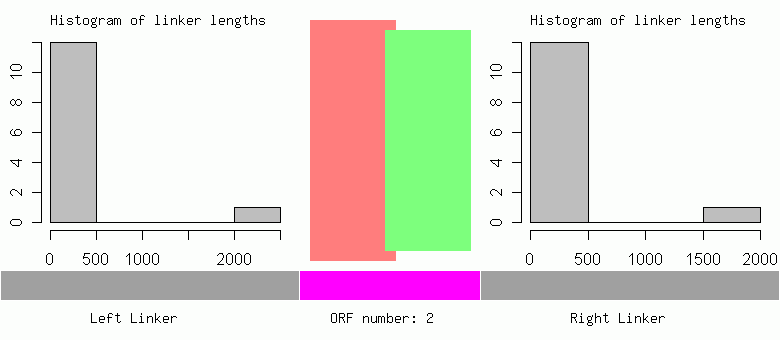


### Family: IS*200*/IS*605*, Group: IS*607*


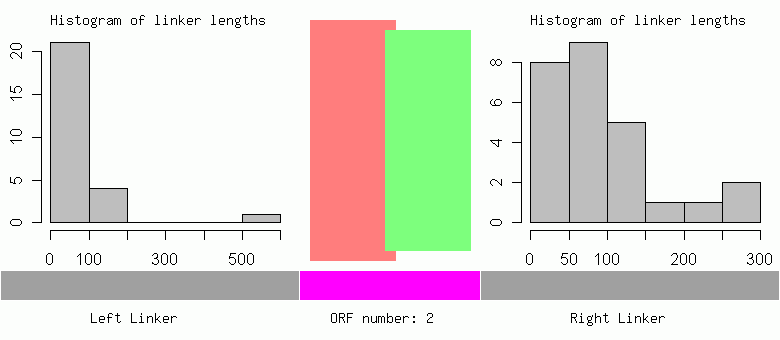


### Family: IS*200*/IS*605*, Group: IS*608*


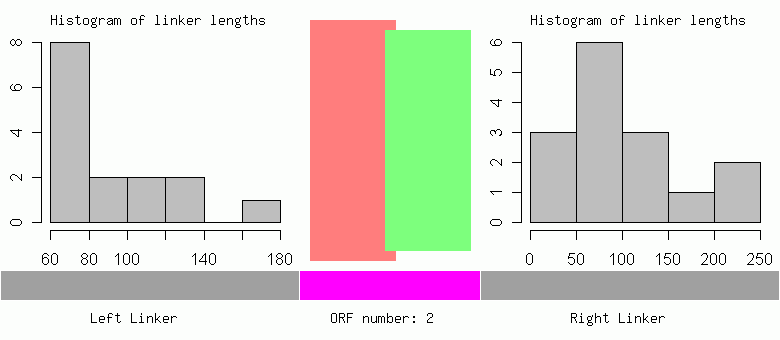


### Family: IS*21*, Group: -


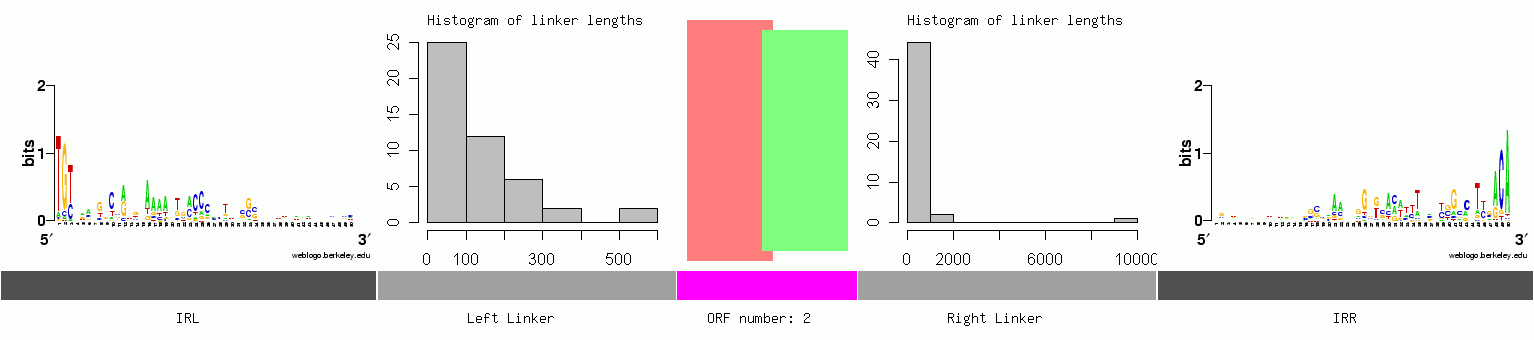


### Family: IS*256*, Group: -


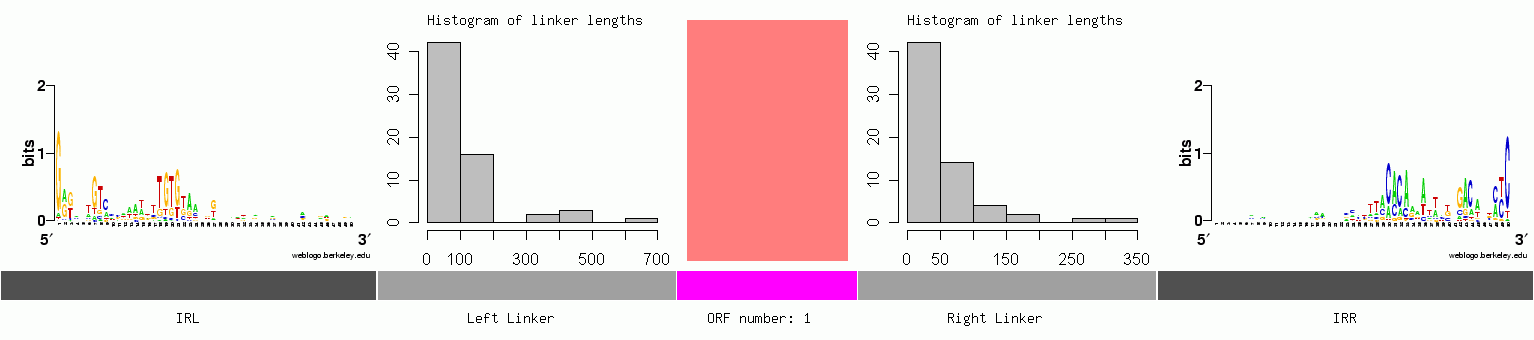


### Family: IS*3*, Group: -


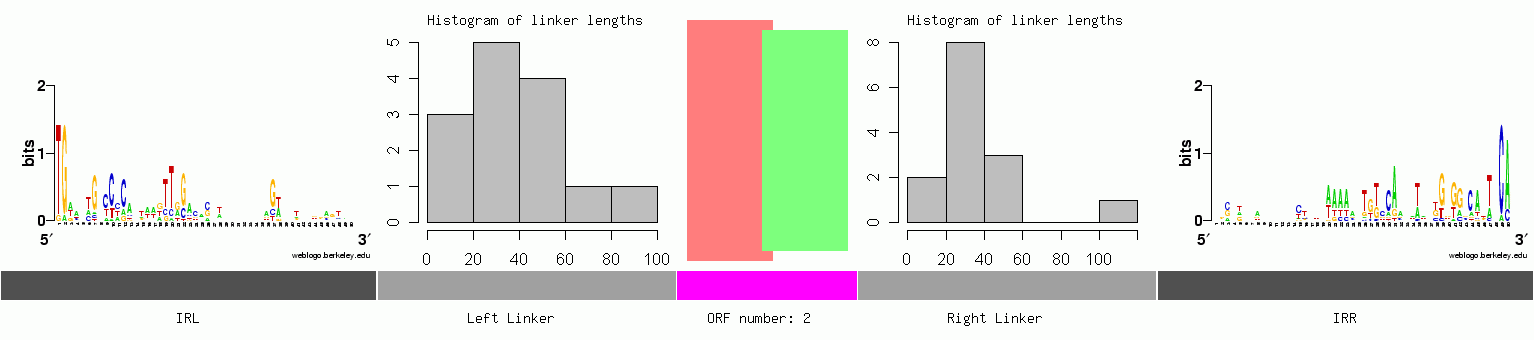


### Family: IS*3*, Group: IS*150*


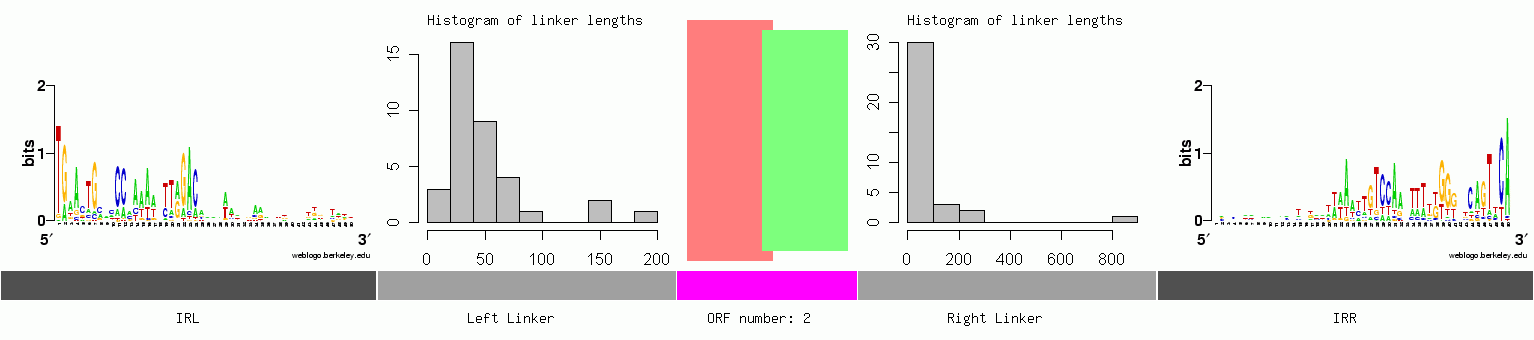


### Family: IS*3*, Group: IS*2*


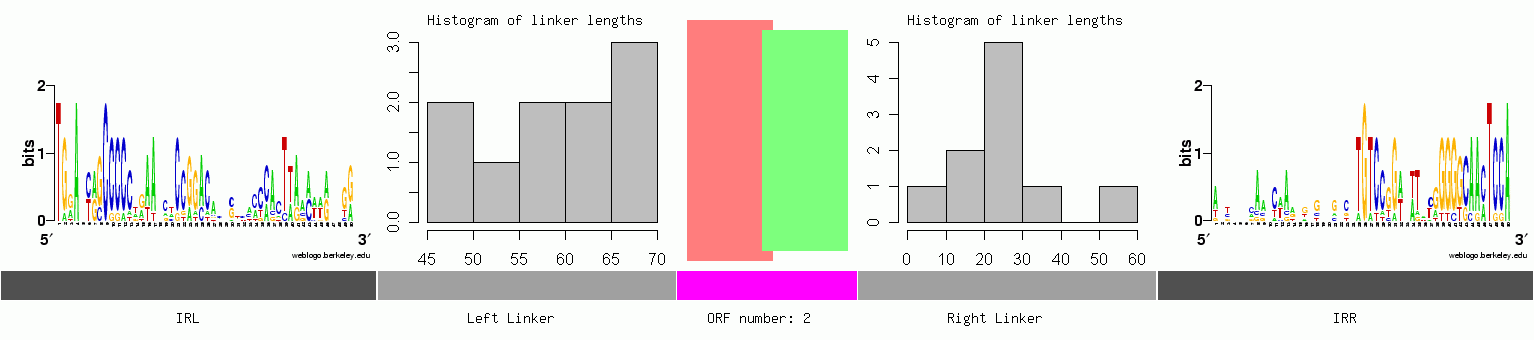


### Family: IS*3*, Group: IS*3*


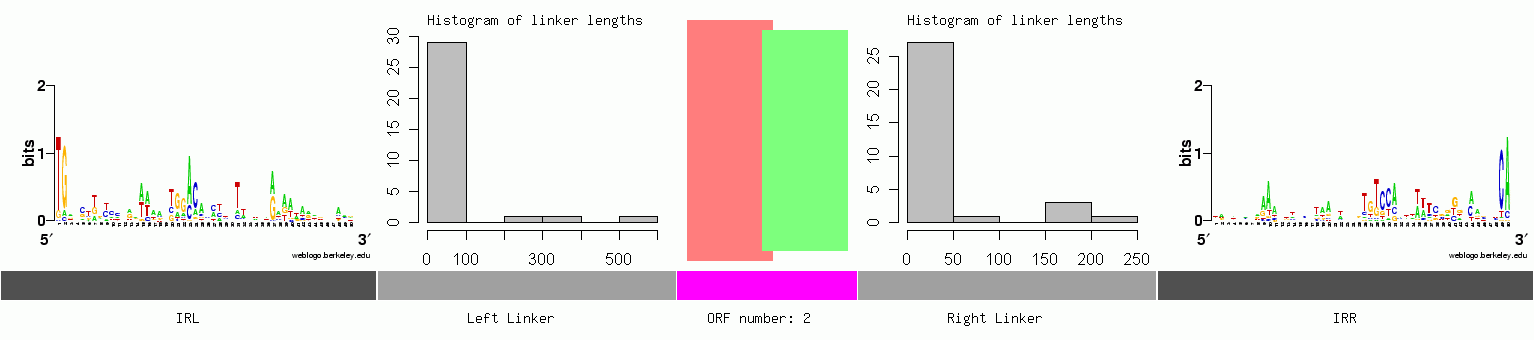


### Family: IS*3*, Group: IS*407*


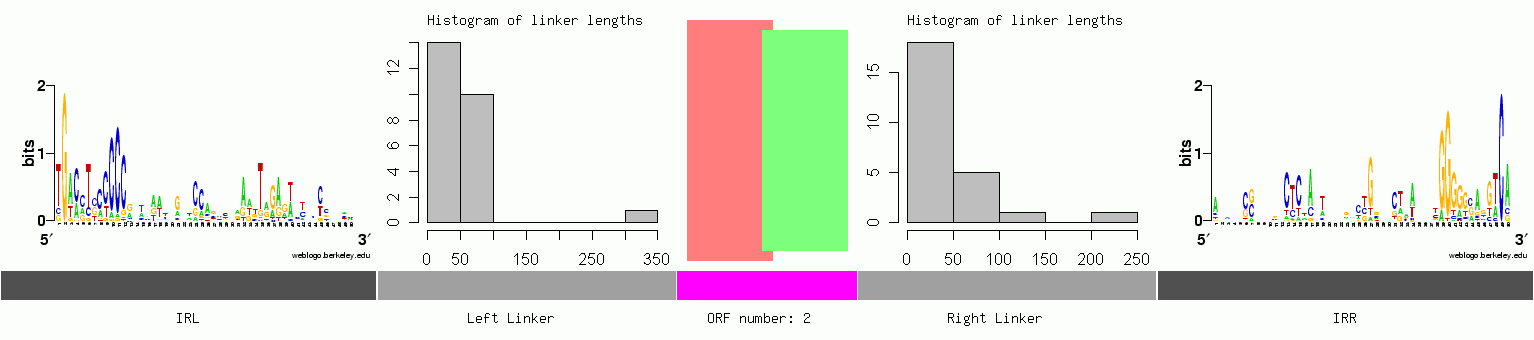


### Family: IS*3*, Group: IS*51*


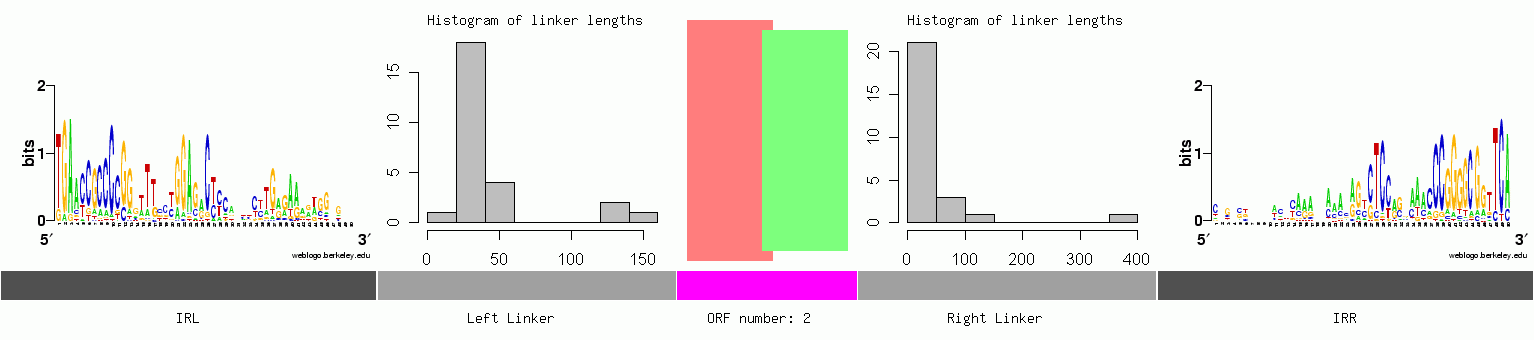


### Family: IS*30*, Group: -


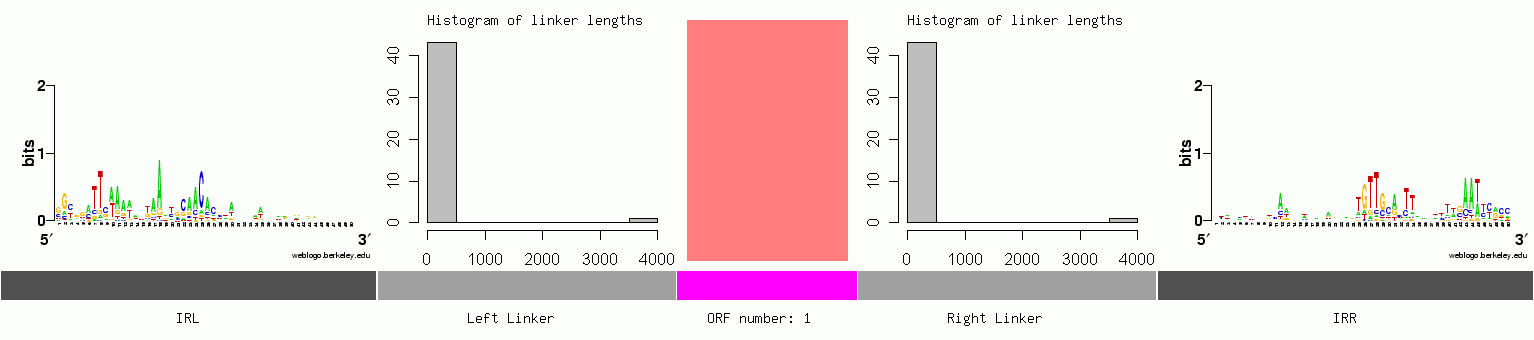


### Family: IS*481*, Group: -


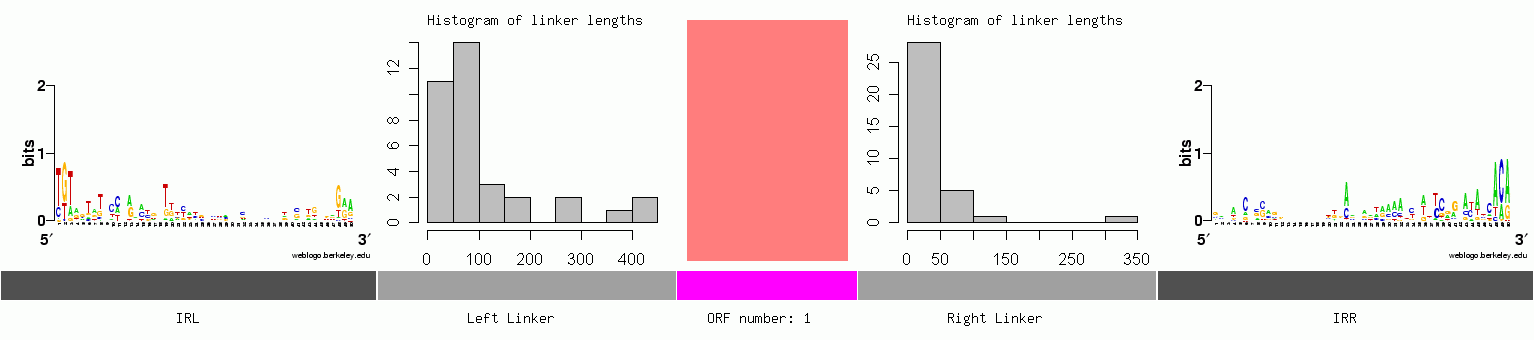


### Family: IS*6*, Group: -


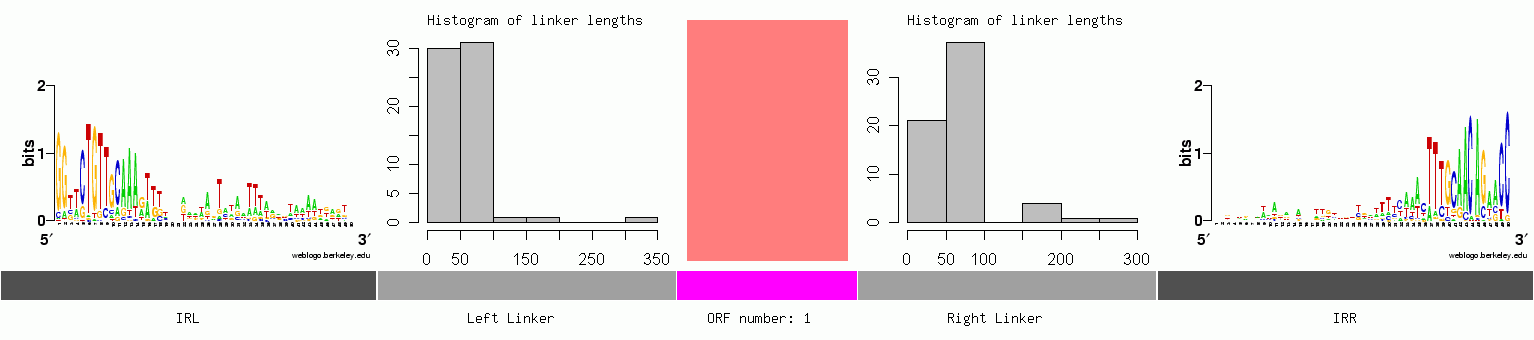


### Family: IS*4*, Group: -


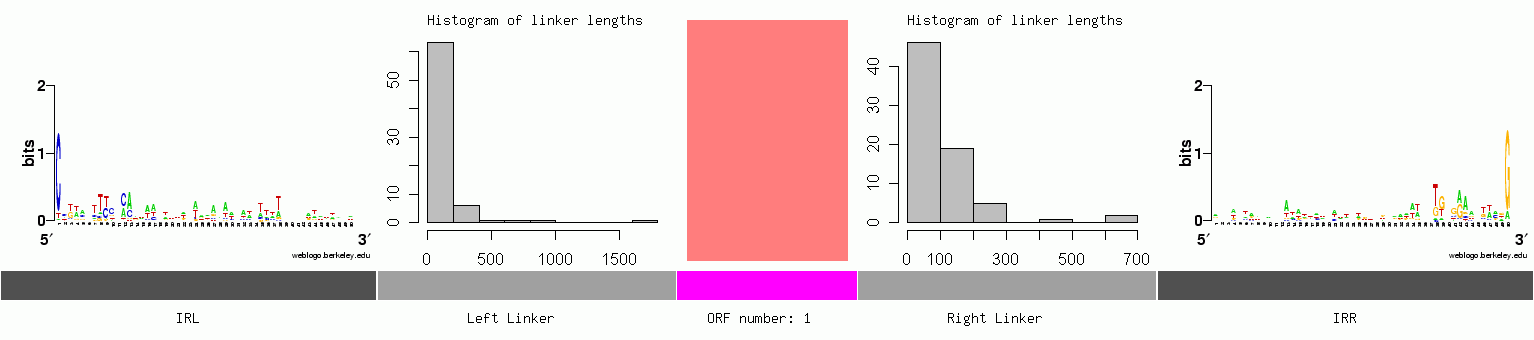


### Family: IS*4*, Group: IS*231*


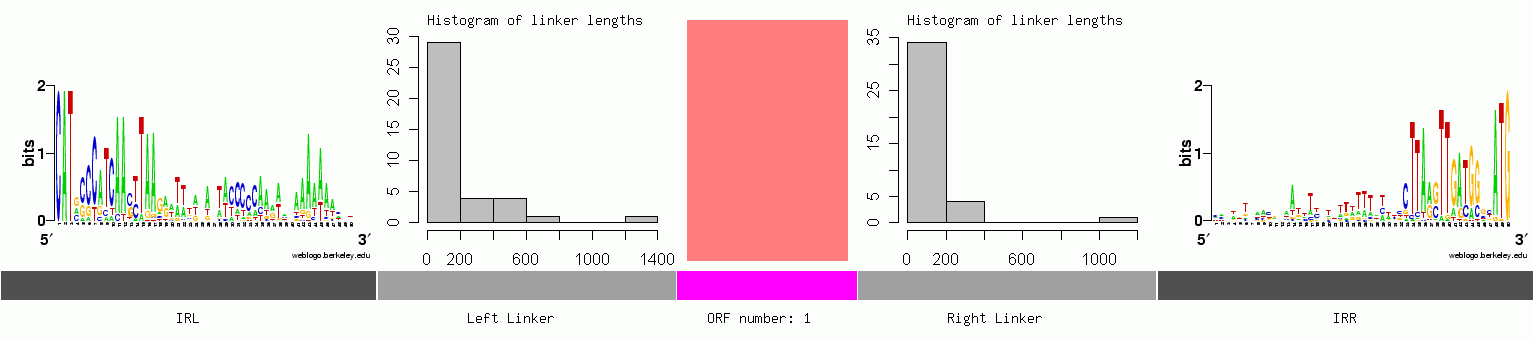


### Family: IS*4*, Group: IS*4*


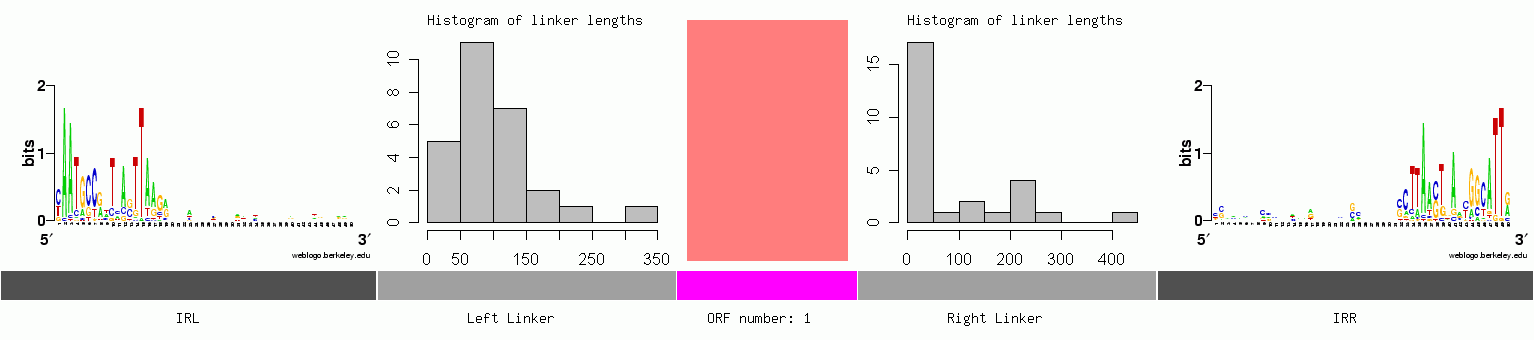


### Family: IS*4*, Group: IS*4Sa*


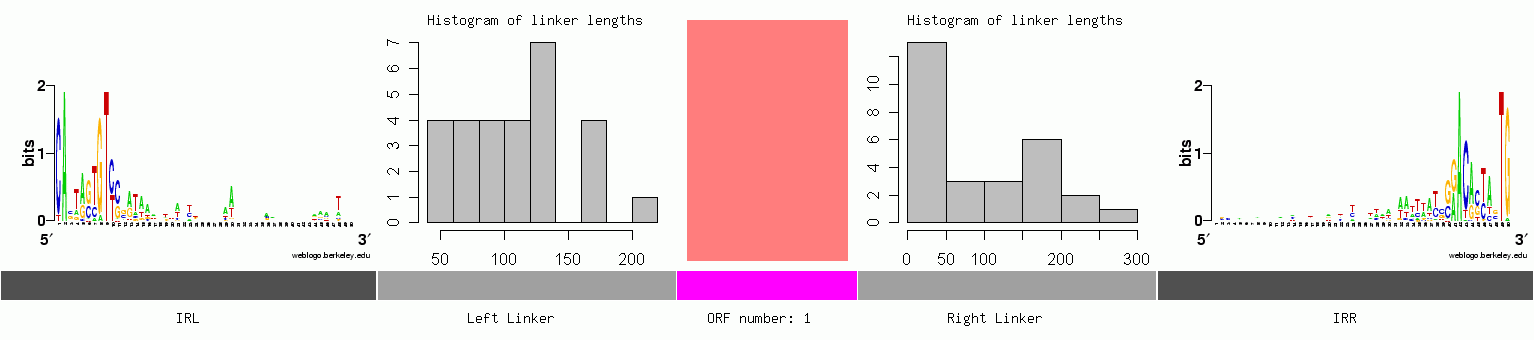


### Family: IS*4*, Group: IS*H8*


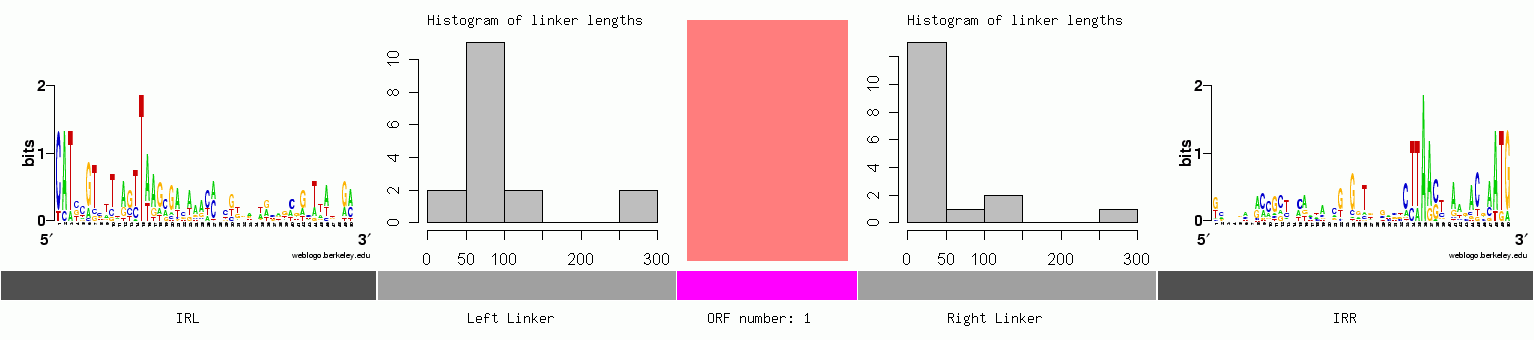


### Family: IS*5*, Group: -


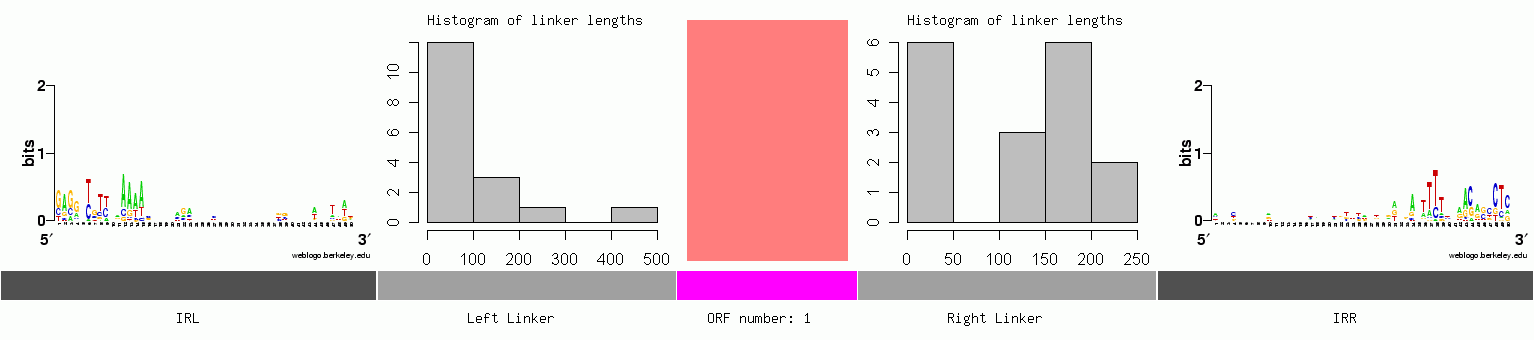


### Family: IS*5*, Group: IS*1031*


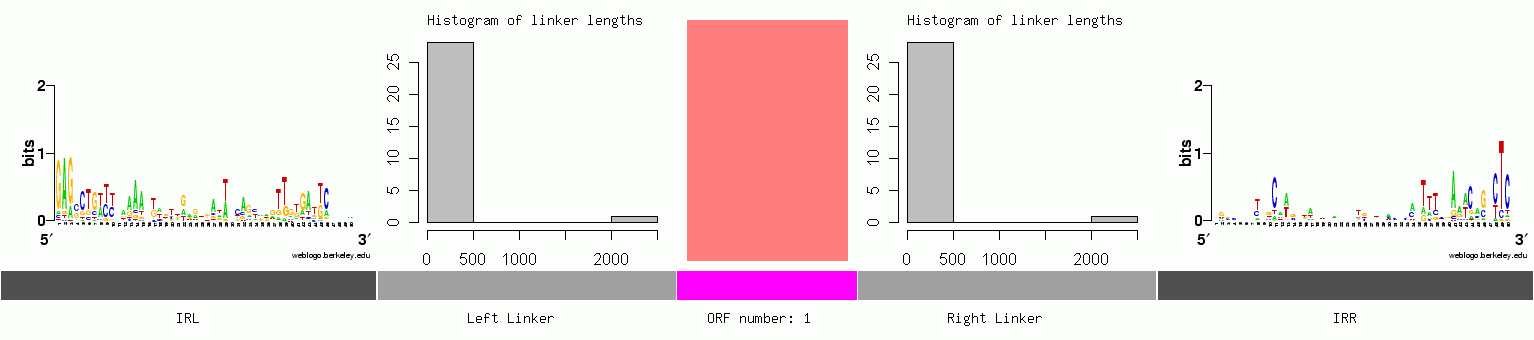


### Family: IS*5*, Group: IS*427*


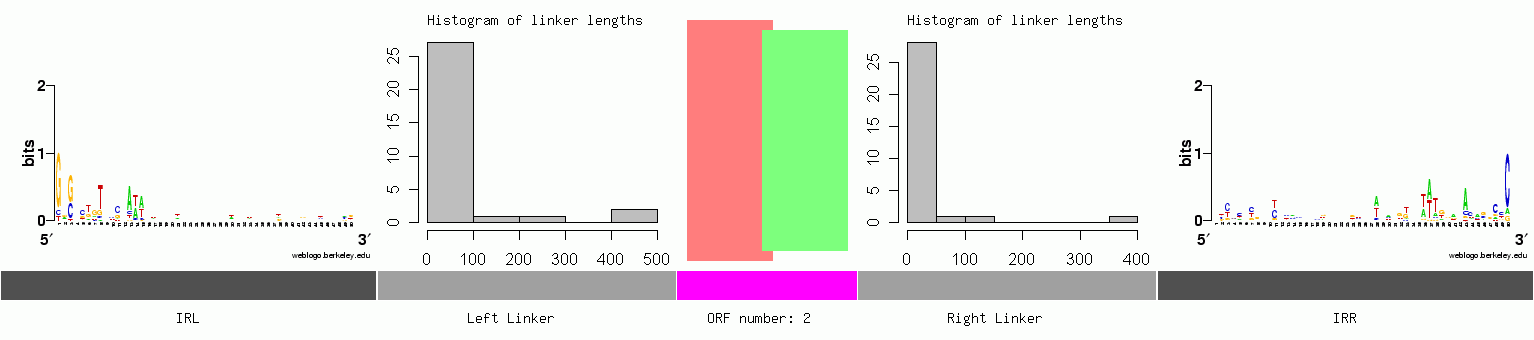


### Family: IS*5*, Group: IS*5*


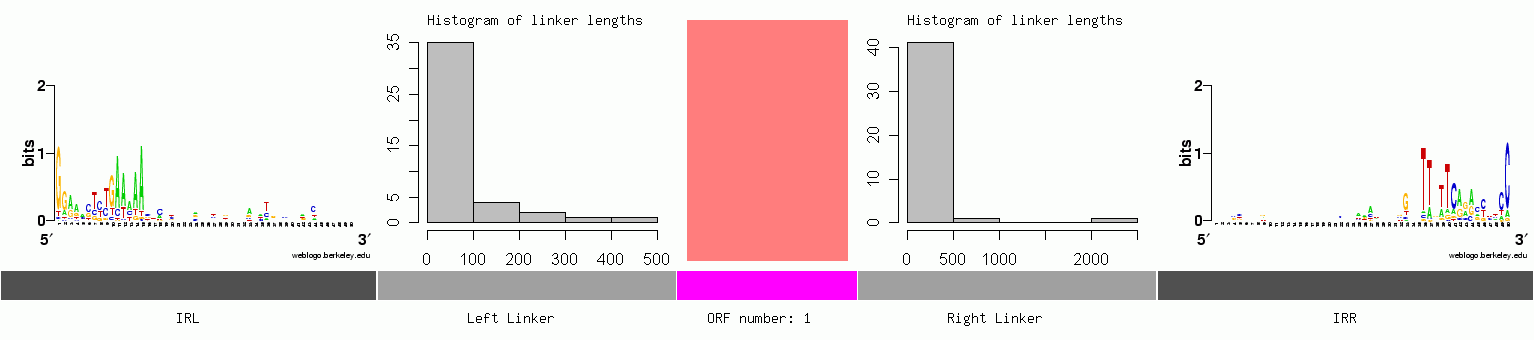


### Family: IS*5*, Group: IS*903*


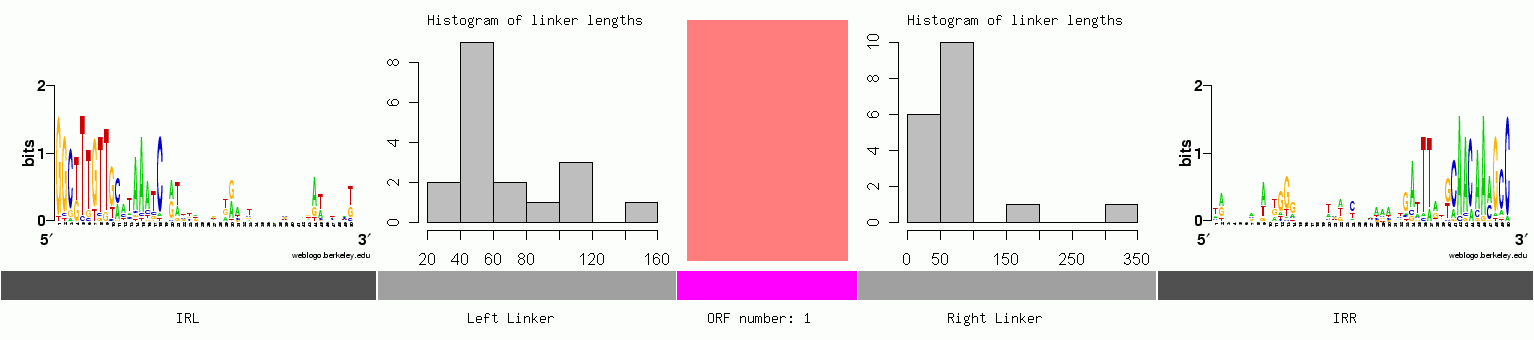


### Family: IS*5*, Group: IS*L2*


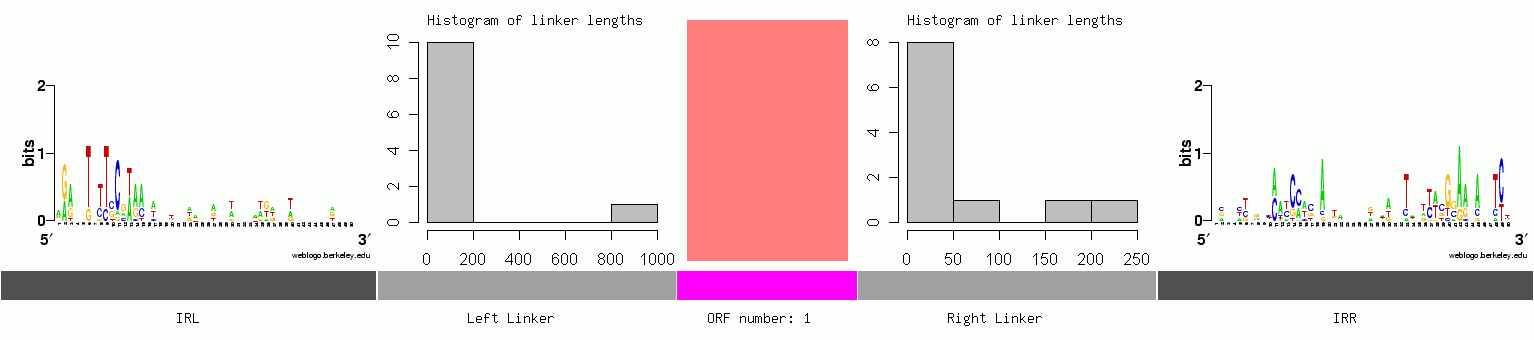


### Family: IS*630*, Group: -


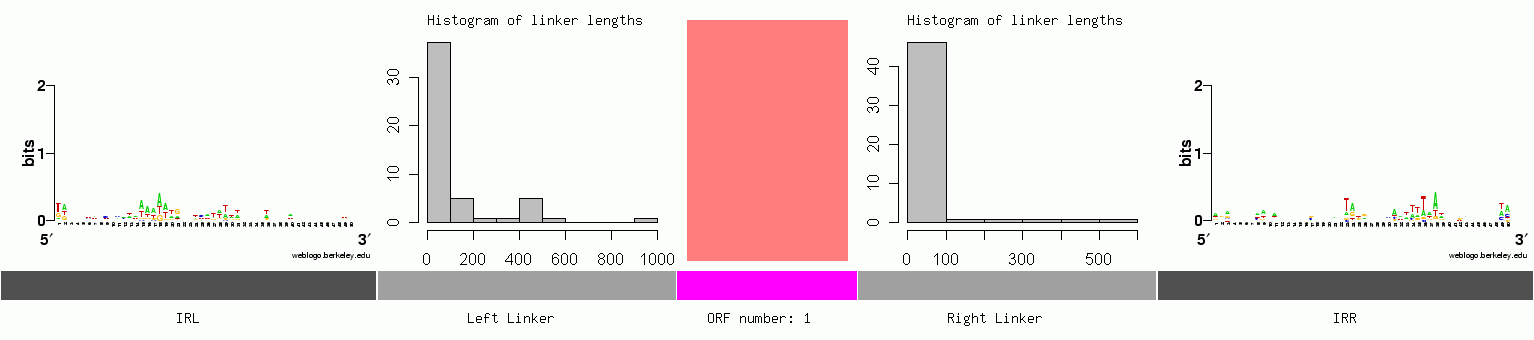


### Family: IS*66*, Group: -


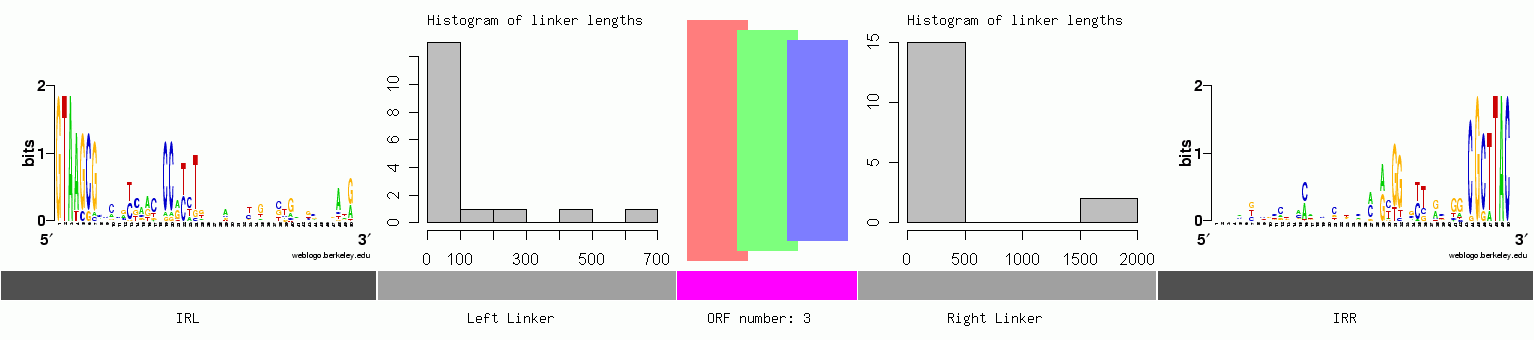


### Family: IS*982*, Group: -


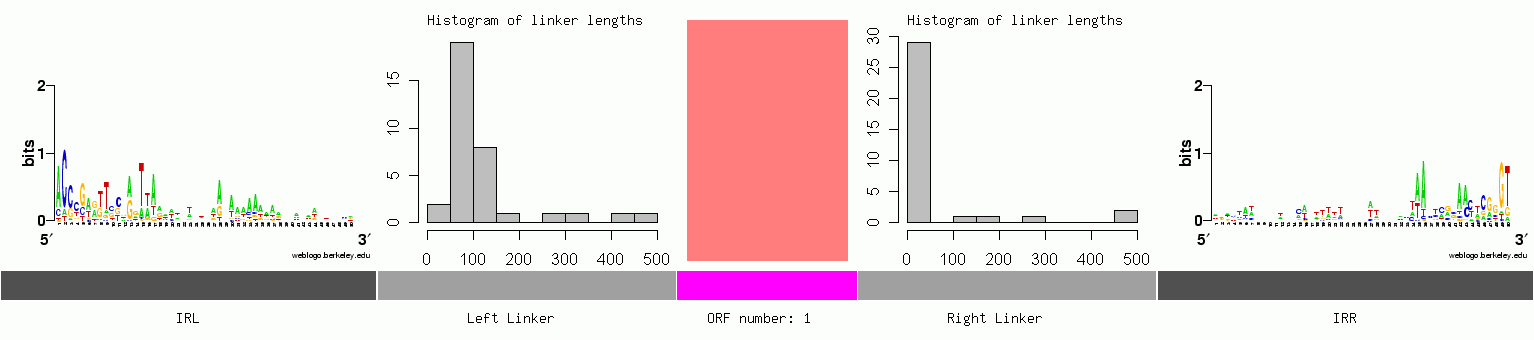


### Family: IS*As1*, Group: -


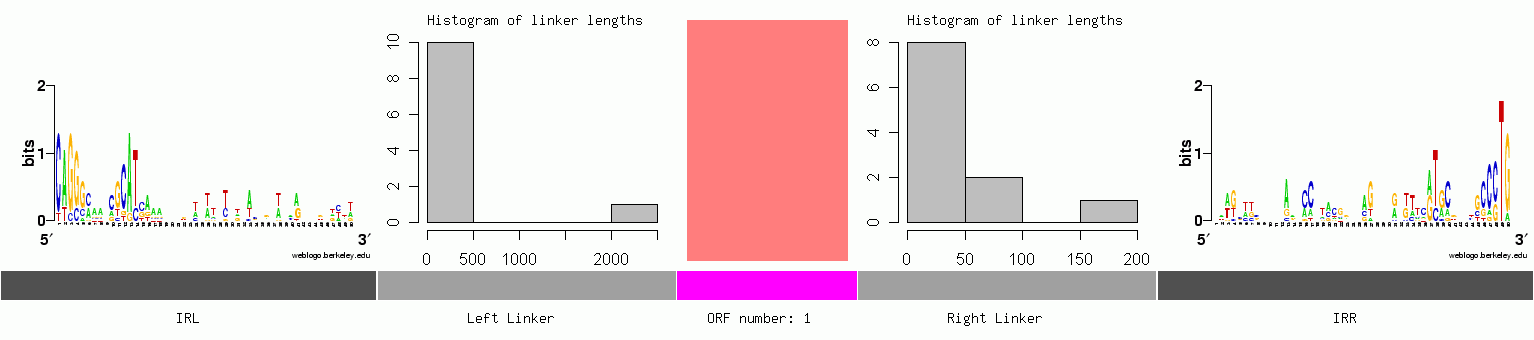


### Family: IS*L3*, Group: -


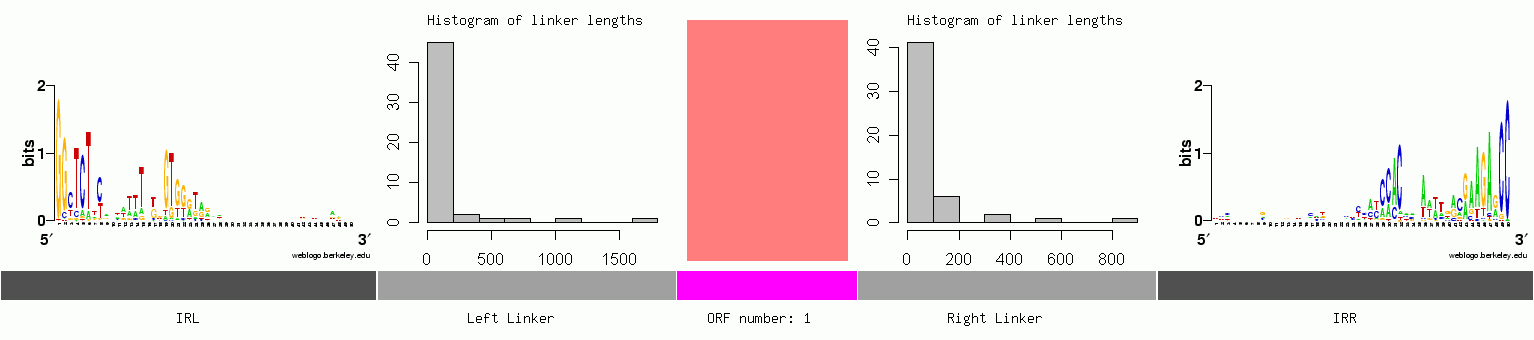


**Supplementary figure S2.** The histograms of the conservation percentages (a) between pairs of IS elements in the same groups and in different groups, and (b) between pairs of IS elements in the same families and in different families.


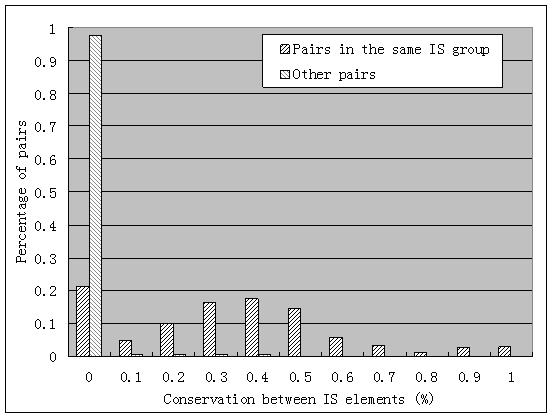

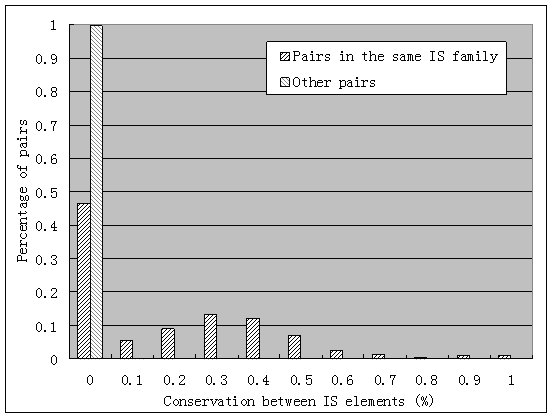


(a) (b)

**Supplementary figure S3.** The upstream and downstream flanking sequences of a predicted full copy of a TIR IS element in the target genome sequence are retrieved for TIR signal scanning.


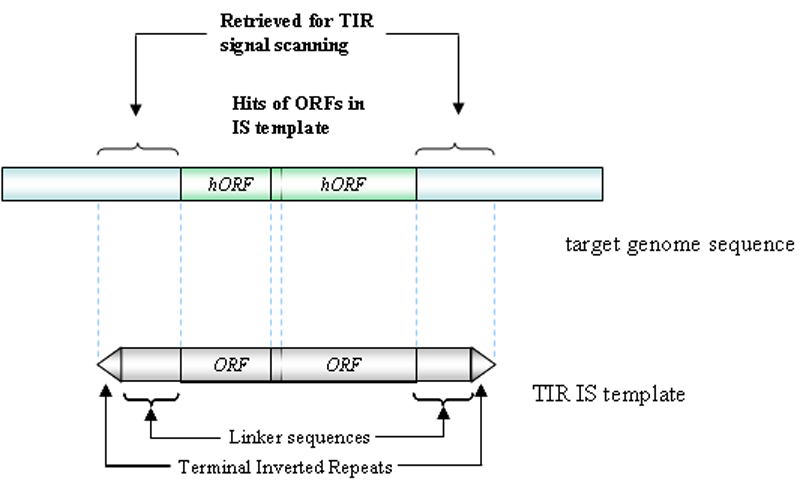

Supplement: Additional File 2 — Supplementary Figures S1–S3. Supplementary Figure S1 shows the schematic profiles of all the 36 IS groups with at least 10 members. Supplementary Figure S2 (a) and (b) compare the conservation percentages between pairs of IS elements at the levels of IS groups and families, respectively. Supplementary Figure S3 shows how to retrieve TIR signals for a predicted full copy of a TIR IS element. [file 1471-2164-9-36-S2.doc]
